# Supplementary material for: Development of a Novel Anti-CD44 Variant 6 Monoclonal Antibody C44Mab-9 for Multiple Applications against Colorectal Carcinomas
Source: Int J Mol Sci. 2023 Feb 16;24(4):4007. doi: 10.3390/ijms24044007 (PMC9965047; doi:10.3390/ijms24044007)
Supplement: Supplementary file 1 [file ijms-24-04007-s001.zip › ijms-2174726-supplementary.pdf]

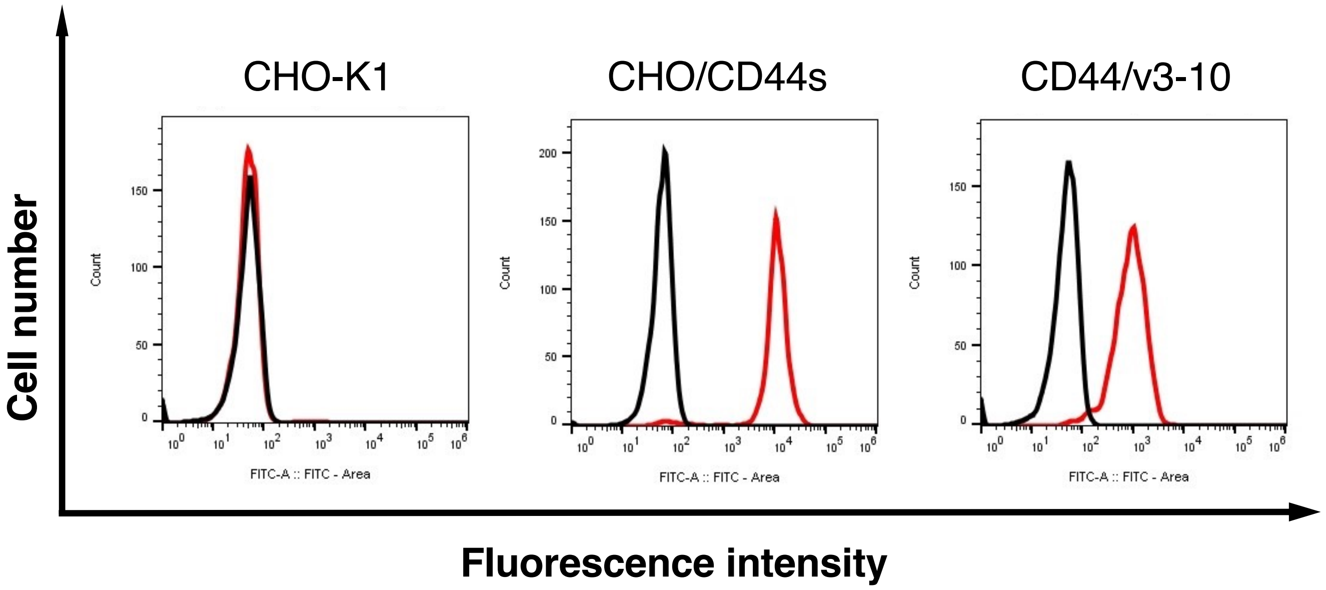

Figure S1 Conformation of the recognition of CHO/CD44s and CHO/CD44v3-10 by C_{44}Mab-46 by flow cytometry. CHO-K1, CHO/CD44s, CHO/CD44v3-10, were treated with 10 μg/mL of C_{44}Mab-46, followed by treatment with Alexa Fluor 488-conjugated anti-mouse IgG (Red line). The black line represents the negative control (blocking buffer).
